# Modeling the Bioenergetics and Life History Traits of *Chironomus riparius*–Consequences of Food Limitation

**DOI:** 10.3390/insects15110848

**Published:** 2024-10-30

**Authors:** Evridiki Klagkou, Andre Gergs, Christian U. Baden, Konstadia Lika

**Affiliations:** 1 Department of Biology, University of Crete, Voutes University Campus, 70013 Heraklion, Greece; biop1070@edu.biology.uoc.gr; 2 Bayer AG, Crop Science Division, Alfred-Nobel Straße 50, 40789 Monheim, Germany; andre.gergs@bayer.com (A.G.); christian.baden@bayer.com (C.U.B.)

**Keywords:** dynamic energy budget theory, holometabolous insects, aquatic insects, *Chironomus riparius*, biphasic larval growth

## Abstract

Chironomids have a number of characteristics that make them a useful group for investigating the impact of environmental and chemical stressors on their life cycle stages. It is crucial to first understand sensitivities to environmental factors and provide a basis for interpreting the results of toxicity tests. We focused on *Chironomus riparius*–one of the most studied species in aquatic toxicity tests—to understand the changes during the larval stage under conditions of food abundance and limitation. We developed a model based on Dynamic Energy Budget (DEB) theory, a framework to capture the entire life cycle of an individual under varying food and temperature conditions. Available information from this study and the literature pointed out that the first three larval instars are immature and the fourth larval instar is mature, during which the organism saves, in two phases, energy for essential processes occurring during the subsequent non-feeding stages. The model can successfully predict the observed prolonged fourth instar duration under food limitation, the times of life history events (e.g., pupation and emergence), and egg production. This model has the potential to be integrated with toxicokinetic–toxicodynamic models to study the effects of toxicants on a variety of biological traits.

## Simple Summary

Aquatic insects are commonly used to monitor freshwater quality and study the effects of chemicals. Recently, mathematical models that analyze chemical exposure have become more popular. Factors like food availability and water temperature greatly affect the growth and survival of insects. It is important to understand their life cycle and how they respond to environmental changes. Mathematical modeling helps us understand how environmental stress affects organisms’ life cycles. In this study, we present a mathematical model for the harlequin fly, a non-biting midge, based on existing research and new data. The model effectively predicts various bioenergetic and life history outcomes, making it useful for studying environmental stress.

## 1. Introduction

Chironomidae, commonly known as non-biting midges, are found in all types of freshwater ecosystems at high abundances [1,2]. As holometabolous insects, they experience complete metamorphosis. Their life cycle begins as an egg, which then hatches into larva. The larval stage consists of four instars, where each instar is separated from the next by a molt. After the last molt, the insect becomes an immobile non-feeding pupa which emerges as an adult (imago). The immature (egg, larval, and pupal) stages are aquatic and the imago stage is terrestrial [3]. Chironomids are considered r-strategists, meaning that they reach a smaller body size but have high reproductive rates [4,5,6,7]. Most species have one or two generations per year [8]. During their larval stage, they are exposed to changes in their environment, such as different temperatures, pH levels, nutrient availability, and substrate content [9]. When they are exposed to low food levels, they tend to increase the duration of the larval stage, while they manage to keep the number of eggs produced at a high level despite their low survival [10,11,12,13,14]. Chironomids have been used for years as bioindicators of water temperature (by the subfosil remains of larvae) and quality (specifically the chemical content in the water), with the amount of studies increasing continuously for decades [1,2,15,16,17].

In this study, our focus was on *Chironomus riparius*, commonly referred as the harlequin fly, which is a widely spread species mostly found in Europe and North America [18] in both lotic and lentic environments [11]. Its life cycle consists of the aquatic egg, larva (four instars), pupal stage, and a terrestrial imago [19]. *C. riparius* is one of the most used chironomid species in aquatic toxicity tests [19,20,21,22,23,24,25,26,27,28] and in mathematical modeling. Energy-based models have been developed to investigate effects of food availability and temperature on the growth and reproduction of *C. riparius* [11,29,30] and population models to study the effects of sediment-exposed scenarios [12,31,32]. Pery et al. [33,34] adapted a growth model to link food availability and toxicity, and Mafin et al. [35] used a dynamic population model to predict the effects of long-term toxicant exposure on *C. riparius*. Recent studies use toxicokinetic–toxicodynamic models (TKTD) to study the effects of various toxicants on *C. riparius* [36,37,38,39].

The response of the species to changes in food availability and temperature has been extensively studied both experimentally and theoretically. However, there are still unexplained life cycle characteristics such as triggers of the prolonged duration of the last larval instar under food limitation [12]. Pery et al. [11,31] proposed a model to link feeding with growth and reproduction assuming that growth continues till a maximum size is reached, with emergence occurring a few days later. In their model, the mean number of eggs per mass was described as a linear function of food availability. The additional time until emergence reached was estimated for each feeding level. In their approach, the time interval between reaching the maximum size and emergence was obtained empirically and thus cannot be extrapolated to other feeding levels. Moreover, growth and reproduction are not simultaneously linked to food intake and mass balance is not made. Using Dynamic Energy Budget (DEB) theory [40], one can quantify various metabolic processes within a single framework, making use of mass and energy balance under varying food and temperature conditions. The DEB approach connects, in one framework, multiple endpoints, such as length, weight, reproduction, duration of larval stage, time, and length at pupation, to name a few.

Numerous studies have extensively applied DEB models to various organisms, including fish, mammals, birds, molluscs, reptiles, and arthropods (see https://debportal.debtheory.org/docs/DEBpapers.html (accessed on 22 October 2024) for an extensive list of publications). There are, however, few papers for insects that fully developed and analyzed the properties of the underlying model, with the study of Llandres et al. [41] being the only analytical presentation of a DEB model for a holometabolous insect, applied to endoparasitic wasp *Venturia canescens*, and that of Klagkou et al. [42], which proposed models for stink bugs and applied them to *Euschistus heros* and *Nezara viridula*. The Add-my-Pet (AmP) database [43,44] includes a few additional insect species that are less thoroughly analyzed. The AmP database is an open-access, online collection of referenced data on animal energetics, including DEB parameters and traits. It contains eco-physiological data for over 5116 animal species (as of 22 October 2024) from all major phyla, but less than 1% belong to the insecta class. The DEB parameters and traits generated from DEB models for most taxa have been tested and validated for particular species and compared in an eco-evolutionary context (e.g., [45,46,47,48]). However, the DEB modeling framework for insects is still under development and requires further refinement. Most of the DEB models for insects in the AmP collection [44] predict a decreased duration of the nymphal/larval stage when food is limited. This prediction, however, is not supported by data for *C. riparius* [12] or for other species, including *Onthophagus taurus* [49], *Aedes aegypti* [50], and *Episyrphus balteatus* [51]. This prediction is due to the stage transition assumption and it is not featured by models for other taxa. A model for *C. riparius* already existed in the AmP database [52], including several types of datasets for its parametrization. That version also predicted a shorter larval duration in a food limitation scenario. As larval duration is used as an endpoint in environmental risk assessment studies, it is critical that the developed models capture the observed patterns in non-toxicant stress scenarios. Gergs et al. [38] and Koch et al. [39] used the DEB model for *C. riparius* that already existed in the AmP database [52] and proposed ways that result in increasing larval duration under low food availability. While their ideas are worth studying, a detailed description of the *C. riparius* life cycle is needed that take into account existing knowledge and address the limitations identified in [38].

The aim of this study was to understand the changes during the larval stage of *C. riparius* under conditions of food abundance and limitation; in particular, to understand the trigger(s) for the onset of the pupal stage. To this end, we modified the current modeling approach for holometabolous insects [41,53] using detailed information and data available from the literature, supplemented with growth and reproduction data at various food levels produced in this study. Weight measurements, in addition to length ones, were needed to better inform the model structure and parameter estimation since the former is a more accurate measurement for insects. To elucidate the trigger(s) for pupation, we tested various triggers and explored how the modified models predict larval duration and egg production under a range of food levels. We concluded that two triggers were needed to achieve the observed prolonged duration of the fourth larval stage without the number of eggs increasing or remaining unchanged under low food levels: one trigger ensuring adequate energy to produce eggs and the other ensuring enough energy to survive the non-feeding stages of pupa and imago. The proposed modifications aim to improve the predictive power and applicability of the model under food limitation, which then could be a valuable tool for investigating the effects of environmental and chemical stressors. The generality of the DEB modeling framework would allow its use for other Chironomidae and holometabolous insects with similar life cycle.

## 2. Materials and Methods

### 2.1. The DEB Model for Chironomus riparius 

A variety of typified models were derived from DEB theory to incorporate the complexities of the various species’ life stages [54]. The majority of insect species are modeled using the h-models, which assume metabolic acceleration [55] for part of their life cycle; a cease of growth after emergence; filling the reproduction buffer in the last feeding stage before emergence since the imago’s diet typically lacks macro-nutrients and in some cases they do not eat at all; and include the pupa stage for holometabolous insects [54]. The proposed model for *C. riparius* is based on the “hax” model [53], with some modifications which are explained below. Table 1 provides a summary of the model equations, while detailed descriptions are available in Tables S4 and S5 of the Supplementary Materials.

The model is based on physiological rules to quantify the energy derived from food and its use for maintenance, growth, and maturation or reproduction. Growth is measured in terms of an increase in structural body mass. Maturation is the process of allocating energy to increase the organism’s complexity, signifying its progress through the distinct life stages. Once the organism reaches reproductive maturity, energy is stored for reproduction purposes. Maintenance refers to the energy allocated to keep the organism alive (somatic maintenance) and to maintain the current state of maturity (maturity maintenance). An organism is described with four state variables: reserve (*E*), structure (*V*), energy invested for reproduction ($E_{R}$), and energy invested for maturation ($E_{H}$).

**Figure 1:**
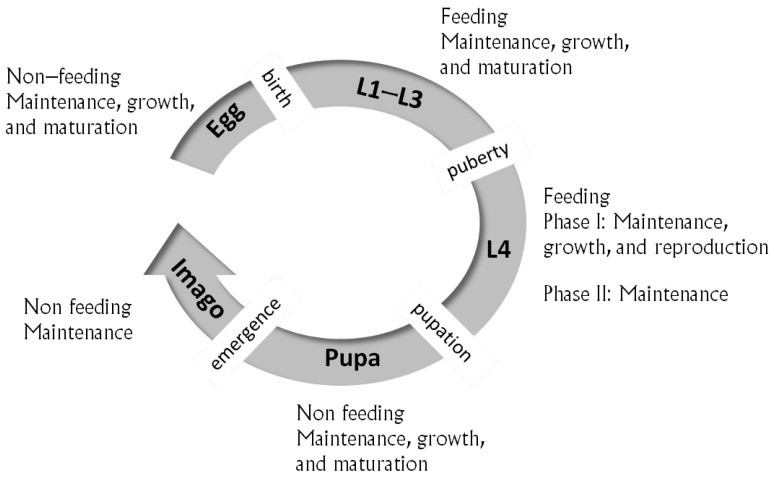
Schematic presentation of the proposed hax model for *C. riparius*, including the morphological life stages with their functional processes and the life history events. The life cycle includes the aquatic stages of egg, larva, and pupa and the terrestrial imago stage. The larval stage consists of four instars L1–L4, with puberty occurring during the molt from L3 to L4. The last larval instar is divided into two phases with distinct functional processes. The imago lays eggs using the energy allocated into the reproduction buffer during the L4 instar.

The model describes the entire life cycle of the holometabolous *C. riparius* (Figure 1), starting as an embryo within an egg. Upon hatching (birth), it enters the larval stages with distinct functions before pupating. Finally, it emerges from the pupa as an imago. The organism alternates between feeding (larva) and non-feeding (embryo, pupa, and imago) stages. During the feeding stages, the organism derives energy and nutrients from food, which are converted into reserves to provide the energy for the metabolic processes. During the non-feeding stages, metabolism is fueled from the stored reserves; embryos use the energy stored in the egg. A fixed fraction κ of the mobilized energy is allocated to the somatic branch of metabolic processes (somatic maintenance and growth) and the remaining 1−κ is directed toward the maturation/reproduction branch, including maturity maintenance. This energy allocation scheme is termed κ-rule [40].

Embryo and pupa, which do not feed, use the stored energy to mature, grow, and pay maintenance costs. Larva, which feeds, uses energy mobilized from the reserve to increase its state of maturity (the immature larval stage) or allocates it to reproduction (the mature larval stage), growth, and maintenance. The imago, which does not feed, uses the reproduction energy stored during the mature larval stage to lay eggs. The transition from one stage to another occurs when specific thresholds are reached. In particular, the transition from the embryonic stage to larval stage (termed birth or hatching) occurs when the maturity level reaches a specified threshold $E_{H}^{b}$ (i.e., $E_{H}=E_{H}^{b}$) and during the transition from the immature to mature larva (termed puberty) when $E_{H}=E_{H}^{p}$. For *C. riparius*, it is assumed that the immature larval stage includes the first three instars (L1–L3) and the mature larval stage includes the L4 instar, with puberty occurring during the last molt from the L3 to L4 instar. This assumption is supported by various experimental evidence. Planello et al. [56] showed that the expression patterns of genes in the ecdysone pathway indicate that vitellogenin expression is induced in the fourth instar larvae. This suggests that vitellogenin production occurs during this developmental stage to ensure sufficient energy reserves in the next generation’s oocytes and embryos, given that *C. riparius* adults do not feed. Experimental evidence also shows differences between sexes in growth [11,57] and energy reserves [58] during the fourth larval instar.

After puberty and during the fourth larval stage, the mature larva does not invest in maturation, with the maturity level remaining at $E_{H}=E_{H}^{p}$. This means that an alternative trigger for pupation should be investigated. Since *C. riparius* does not feed during the pupa and adult stage [59], it should accumulate energy during the fourth larval stage to be used to make eggs in the imago stage and ensure sufficient energy reserves to survive [5,10]. The existing h-typified DEB models for insects ([53], Chapter 7) assume that the onset of the pupal stage occurs when the reproduction buffer density, $\left[E_{R}\right]=\frac{E_{R}}{V}$, reaches a certain threshold $\left[E_{R}^{j}\right]$. This trigger for pupation was also assumed in [41] for modeling the life cycle energetics of the holometabolous endoparasitic wasp *Venturia canes*. Their model differs from the proposed model in their assumption that the entire larval stage is mature, meaning that it invests in reproduction and the maturity level is constant and equals that at birth. This allocation scheme, where the reproductive buffer is filled during the entire larval stage, is modeled by the hex model [54]. For holometabolous insects, reaching a species-specific critical weight is a crucial prerequisite for initiating metamorphosis. Reaching the critical weight ensures that the insect has accumulated sufficient energy reserves to support the extensive cellular reorganization that occurs during the subsequent non-feeding pupal stage, as well as to survive to complete its reproductive cycle. To meet this condition, in addition to the reproduction buffer density trigger for pupation, we assume that the reserve density, $\left[E\right]=\frac{E}{V}$, should reach a critical value, independent of the food availability. This critical value is assumed to be the maximum reserve capacity, $\left[E_{m}\right]$, i.e., the reserve density at abundant food, defined as the ratio of the surface-area-specific maximum assimilation rate, $\left\{{\dot{p}}_{Am}\right\}$, and the energy conductance, $\dot{v}$. Therefore, a larva at the fourth instar undergoes biphasic growth (Figure 1): in Phase I, larva allocates energy to growth, reproduction, and maintenance according to the κ-rule. Upon reaching the reproduction buffer density threshold, $\left[E_{R}\right]=\left[E_{R}^{j}\right]$, larva enters Phase II, where it stops allocating energy to increase structural mass and the reproduction buffer. Instead, the energy acquired from food is used to maintain the existing structural mass and maturity and increase the general reserves, *E*. Pupation occurs when the reserve density reaches its maximum capacity $\left[E\right]=\left[E_{m}\right]$. The splitting of the fourth larval instar in two phases is to capture the long instar duration at low food level while keeping the number of eggs produced high [11,60].

Once the larva turns into pupa, the larval structural mass degenerates and the imaginal discs turn into an imago structure. Typically, in a DEB context, there are two modeling approaches for pupa. One approach, as described in [41], assumes that the larval structure gradually decays and fuels the reserve acquired as larva and the other assumes an instantaneous breakdown of structure and conversion into a reserve ([40], Chapter 7.8.1). Since the duration of *C. riparius* pupa is about one day and no data were available on its dynamics to estimate the rate of the degradation of the structure, we adopt the second approach. Like the embryo, the pupa does not feed; it builds a new structure and invests energy to maturation, and both are reset to 0. It allocates the energy according the κ-rule. The pupa does not allocate energy to reproduction but retains the reproduction buffer filled during the larval stage. Once the maturity hits a threshold, $E_{H}=E_{H}^{e}$, the organism emerges to an imago, during which the organism no longer eats, grows, or invests in maturation or reproduction. It pays the maintenance costs and uses the energy stored in the reproduction buffer to lay the eggs. The average number of eggs per female is calculated as $N_{i}=\kappa_{R}\left[E_{R}^{j}\right]V_{j}/E_{0}$, where $\kappa_{R}$ is the reproduction efficiency and $E_{0}$ is the initial energy content of the egg.

In DEB theory, the relationship between surface area and volume is crucial since surface area is relevant for acquisition processes and volume for the maintenance process [40]. This relationship is defined by the shape of the organism. When the organism does not change shape during growth, it is called an isomorph, and the surface area is proportional to the structural volume to the power 2/3. In the case of holometabolous insects and many other animals with larval stages, this assumption does not hold during its entire ontogeny [54]. During part of their life, these animals exhibit metabolic acceleration, i.e., the isomorphic individual temporarily switches to the V1-morphic growth mode, which implies that surface area is proportional to structural volume [55]. In the present model, we assume that the immature larva grows as a V1-morph, while all other stages grow isomorphically. During the acceleration period, the surface-area-specific assimilation rate $\left\{{\dot{p}}_{Am}\right\}$ and the energy conductance $\dot{v}$ increase with the structural length $L\equiv V^{1/3}$. After this period, both parameters remain constant but at a different value from the one before acceleration. Specifically, these parameters are multiplied by the acceleration factor $s_{M}=\max\left(1,\min\left(L,L_{p}\right)/L_{b}\right)$, where $L_{b}$ and $L_{p}$ are the structural lengths at birth and puberty, respectively.

A DEB model for *C. riparius* already existed in the AmP database [44] as a hax-typified model [52]. The proposed model differs from that in two main assumptions. A major modification we applied to the model was the assumption of biphasic growth at the fourth instar. The other assumption concerns the time of puberty specified during the last molt from the L3 to L4 instar. The existing hax model does not explicitly specify this transition from the immature to mature larval stage.

The linking of the state variables of reserves and structure to measured quantities, such as the length and wet weight, was described before in various papers (e.g., [42]). The wet weight of an individual has contributions from the structure, reserve, and energy reserve in the reproduction buffer (for mature larva, pupa, and imago). We assume in this study that the reproduction reserve consists only of dry mass, based on observations that most aquatic insect eggs, since they live in the water, have the ability to absorb water and enlarge once they are laid [61]. This assumption is also supported by the observation that the percentage of dry weight during the fourth instar increases [62].

### 2.2. Incorporating Temperature Effects and Food Availability

Temperature and food availability are the two main environmental drives in the model, converting external conditions into physiological responses of the organism.

The scaled functional response, *f*, quantifies food availability, defined by the Holling type II equation $f\left(X\right)=X/\left(X_{K}+X\right)$, where *X* is the food density and $X_{K}$ is the half-saturation constant and represents the feeding rate as a fraction of the maximum feeding rate achievable by an individual of a particular size eating the same type of food. Its values range between 0 (food deprivation) and 1 (food abundance). Values larger than 1 could be used to account for different diets and experimental protocols.

Temperature affects all metabolic processes via the correction of the rate parameters. The temperature correction factor, $c_{T}$, is assumed to be the same for all metabolic rates and, in this study, depends on three species-specific parameters: the Arrhenius temperature $T_{A}$, the upper boundary of the optimal thermal range $T_{H}$, and the Arrhenius temperature for the rate of decrease at the upper boundary $T_{AH}$. The rate $\dot{k}$ for a process at temperature *T* is obtained as

$$\dot{k}\left(T\right)=\dot{k}\left(T_{ref}\right)c_{T}\left(T,T_{ref},T_{A},T_{AH},T_{H}\right)$$

where $\dot{k}\left(T_{ref}\right)$ is the rate at the reference temperature $T_{ref}$. The full temperature correction function is presented in the Supplementary Material.

### 2.3. Data

To estimate the DEB model parameters, we combined information from the scientific literature with data generated from experiments to supplement the existing data with more valuable insights. A hax DEB model for *C. riparius* was already available from the AmP collection [44], and we used those data for our estimation, supplementing it with the newly generated experimental data. The literature datasets included age at birth, length at puberty, length–weight and time length for both sexes at different food levels [11], time at pupation and time length at different food levels and temperatures [30], initial egg dry weight [63], wet weight at puberty [64], dry weight of the imago [27], time survival under starvation from the previous AmP entry [52], and dry weight–oxygen consumption [62]. As pointed out in [39], imago longevity in many insects is not related to aging, as modeled in DEB theory. We, therefore, ignored the longevity data used in the existing model. Additional data on the duration of instars and time at puberty and pupation from [12] and time since birth at emergence data from [39] were also used.

A description of our experiment can be found in the Supplementary Materials. Briefly, the aim of the experiment was to measure growth and reproduction for various feeding scenarios. Specifically, different concentrations (0.6, 0.3, 0.15, and 0.1 mg/larva/d) of TetraMin^®^ (Melle, NI, Germany) commercial fish food flakes were used. Measurements of length and wet weight were made every 2–5 days from randomly chosen animals. After the animals reached the imago stage, they were transferred and grouped by diet into new dishes with filter paper to collect the eggs.

Lastly, data on time since hatch at emergence at various temperatures from [39] were included as the model validation; these data were not used in the parameter estimation process.

### 2.4. Parameter Estimation

The analytical procedure for estimating the parameters are detailed in [43]. Briefly, the estimation was completed using the downloadable software DEBtool (DEBtool 2024) run in MATLAB (R2024a) [65]. All parameters were estimated simultaneously using the Nelder–Mead simplex method to minimize the symmetric bounded loss function. The estimation was performed by fitting multiple models to multiple datasets. A dataset can be zero-variate (a single data point) or uni-variate (a set of pairs of numbers). In addition, pseudo-data are introduced to increase the identifiability of parameters due to a lack of information in the data and ensure realistic biological parameters [43,66]. They are treated as data with a very small weight coefficient to reduce their influence in parameter estimates, particularly when the real data identify the parameters well. In the case of insects, in addition to typical pseudo-data, we use as pseudo-data the maintenance ratio, *k*, and set it equal to 1 ($k=\frac{{\dot{k}}_{j}}{{\dot{k}}_{M}}$ with ${\dot{k}}_{j}$ and ${\dot{k}}_{M}$ as the maturity and the somatic maintenance rates, respectively). This ensures that the length at specific events remains relatively constant across different food availabilities, a property that is observed in most insects [42].

For each dataset, a constant functional response, *f*, was estimated, except for the data of the present study, where a variable functional response was used. This choice was mainly data driven (see the Supplementary Materials for details). Specifically, food was assumed to be abundant (i.e., f=1) during the early larval stages (L1–L3), followed by a linear decrease to a minimum value (estimated for each dataset) at a specific time, and this remained constant after that point until pupation. By convention, when the feeding conditions are unspecified, the predictions for the zero-variate data are computed at f=1. Consequently, it is expected that the values of *f* for some datasets may be larger than 1 if feeding conditions are better.

Two goodness-of-fit measures were used to evaluate the overall model performance: the mean relative error (MRE) and the symmetric mean squared error (SMSE) [43]. Values of the MRE and SMSE close to 0 mean that the model predictions are close to the data.

## 3. Results

**Figure 2:**
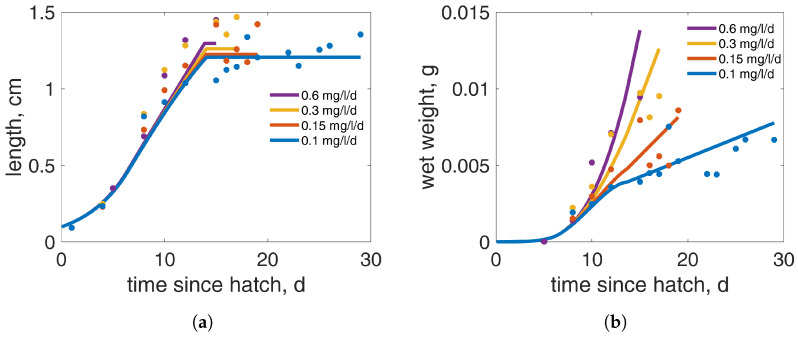
Growth data (points) and model predictions (lines) presented as length (**a**) and weight (**b**) for different feeding TetraMin concentrations (each presented with different colors). Data are from the current study. The experiments were conducted at a constant temperature of 21 °C.

The overall agreement between the data and model predictions was good (MRE = 0.130 and SMSE = 0.029). For comparison, the mean values of the goodness-of-fit metrics for all animals, as well as specifically for the insects in the AmP collection are, respectively, MRE = 0.056, SMSE = 0.014 and MRE = 0.157, SMSE = 0.090, while for that of the previous version of the *C. riparious* model are MRE = 0.140 and SMSE = 0.038 [52]. For the growth data of this study (Figure 2), the model predicted the larval length-at-age trajectories at various food levels well (Figure 2a), except for that at the higher food levels, where the model underestimated the length during the fourth instar. The weight-at-age trajectories, however, fit well at all food levels (Figure 2b). For these datasets, the functional response varied during the larval stage. A preliminary analysis indicated that constant food availability throughout the entire larval stage did not align with the observed weight and length data (see Figure S1 in the Supplementary Materials). Therefore, to better capture the data, as explained in more detail in the Supplementary Materials, a time-variable functional response was assumed, starting at birth with f=1 until puberty, estimated at 6.43 days after hatch, then linearly decreasing until an estimated value $f_{min}$, different for each dataset, is reached at a defined time. In order to limit the number of parameters, the time $f_{min}$ that is reached is assumed to be the same for all datasets, independent of the food condition, which is estimated to be 7.5 days after puberty; times refer to a temperature of 21 °C. The estimated time is close to the transitions from Phase I to Phase II of larva L4 for each food level, which are approximately at 7.4 days post-puberty. However, the timing of a specific event and the functional response, when it is time variable, are linked, and thus it was necessary to estimate the time of the switching point. Differences between the trajectories start around 8 days after hatch when the molt from stage L3 to L4 occurs, but they do not diverge considerably between the experiments before the end of Phase I, due to our assumption that the functional response decreases gradually. The final values of the functional responses for each experiment (0.6, 0.3, 0.15, and 0.1 mg/larva/d TetraMin) were estimated to be 1, 0.58, 0.25, and 0.12, respectively.

**Figure 3:**
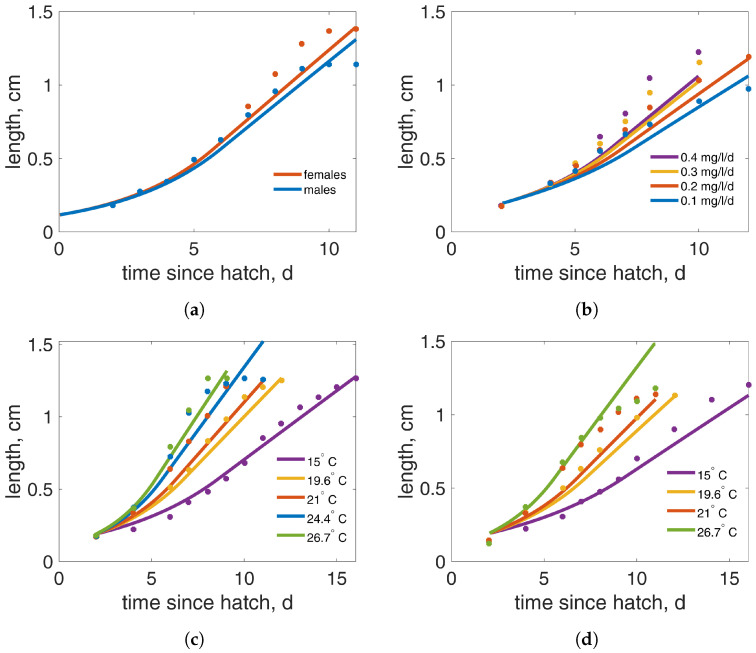
Length-at-age data (points) and model prediction (lines) for (**a**) males and females, (**b**) different TetraMin concentrations, (**c**) different temperatures under ad libitum food, and (**d**) different temperatures under food limitation. Data source: (**a**,**b**) [11] and (**c**,**d**) [30]. The experiments in (**a**,**b**) were conducted at a constant temperature of 21 °C.

The additional length-at-age data from [11,30] were also well captured (Figure 3). Figure 3a illustrates the length at age of males and females, with the length for females being slightly underestimated. This is likely given that the other datasets include measurements from both sexes and the same parameters are used to also fit those data. In this experiment, the food delivered was 1.4 mg/larva/d of TetraMin and the estimated value of the scaled functional response was 1.93. This was expected since this feeding level is larger than what it was considered ad libitum in other studies. The length at age for different concentrations of TetraMin (0.4, 0.3, 0.2, and 0.1 mg/larva/d) are shown in Figure 3b. These data also did not show differences between feeding levels in the initial phase. However, unlike the data in Figure 2, differences in length appear even on day five post-hatching, which is much earlier. This observation and the absence of weight data in this study could not support an assumption of ad libitum food during the first instars. Numerical simulations with a time-dependent functional response, as in the dataset in Figure 2, could not adequately capture the differences between datasets. Therefore, we chose to model these data with constant food availability, and the values of the functional responses for each experiment were estimated to be 1.23, 1, 0.83, and 0.46, corresponding to feeding levels of 0.4, 0.3, 0.2, and 0.1 mg/larva/d TetraMin, respectively. Additional information about the different types of functional response as well as the growth trajectories until pupation is presented in the Supplementary Materials. The length at age for different temperatures was predicted well both in ad libitum conditions (Figure 3c) and during food limitation (Figure 3d). In the ad libitum scenario (Figure 3c), although two concentrations of TetraMin were used (1.2 mg/larva/d for the two highest temperatures and 0.6 mg/larva/d for the three lowest temperatures), we estimated food availability with a single functional response with f=1.50, since initial trials with two different functional responses resulted in almost identical values. For the reduced food experiment (Figure 3d), larvae were fed with 0.2 mg/larva/d of TetraMin and the estimated functional response was f= 0.84, smaller than that estimated for a similar feeding level for the [11] data at $21^{\;\circ }$C. The length measurements from [11,30] were obtained from dead individuals, while in the current study, they were obtained from alive organisms. This may influence the shape of the larva and thus the accuracy of the larval length measurements. To convert structural to physical length, we used different shape coefficients $\delta_{M}$ (Table 2).

**Figure 4:**
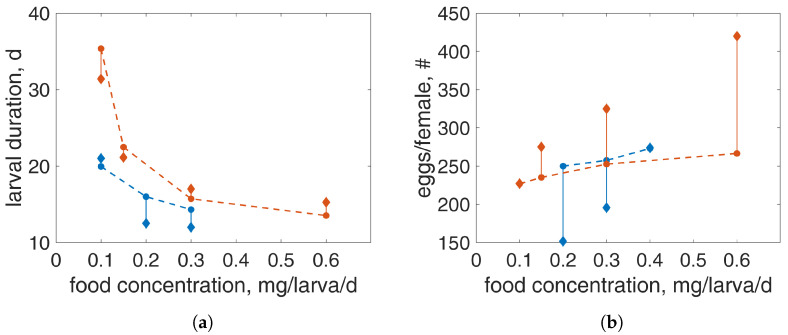
Data (diamonds) and model predictions (circles) for larval duration (**a**) and number of eggs/female (**b**) at different TetraMin concentrations. Vertical lines indicate the error between data and predictions. Data source: current study (orange, (**a**,**b**)), [12] (blue, (**a**)), and [11] (blue, (**b**)). In all experiments, temperature was 21 °C.

Figure 4 illustrates data and model predictions for the larval duration and the number of eggs per female at different concentrations of TetraMin and a constant temperature. Data were compiled from [11,12] and the current study. The model predictions captured the larval duration at all concentrations for both studies well (Figure 4a) while they overestimated the average fecundity per female from the [11] study and underestimated the data from the current study at the highest feeding level (Figure 4b). It is worth pointing out that the number of eggs/female from [11] and the current study do not match, although the experimental conditions are similar. For example, at the feeding level of 0.3 mg/larva/d, Ref. [11] reported 195.6 eggs/female, while in the current study, we reported 325 eggs/female. However, note that for all feeding levels, these two studies do not agree in the number of eggs.

Table 3 lists the remaining zero-variate data with the model predictions, including age, length, and weight at the events birth, puberty, pupation, and emergence. The DEB events were captured very well both in terms of time and growth measurements. By convention, all zero-variate data when feeding conditions are not specified are calculated at f=1. Lastly, the durations of the instars L1–L3 were modeled by partitioning the immature larva phase using Dyar’s Law, which assumes that the length of each instar is proportional to the length of the previous instar (see [42] for details), under the constraint that the transition from L3 to L4 occurs at puberty, i.e., when maturity reaches the threshold $E_{H}^{p}$. The rest of the data used in the parameter estimation with model predictions are given in Figure S3 in the Supplementary Materials. The data include survival under food deprivation and food abundance, oxygen consumption for two temperatures in relation to dry weights, and length as a function of dry weight. For all datasets, model predictions are close to the data, with the exception of oxygen consumption, whereby the model underestimates oxygen consumption for large organisms at a high temperature ($20^{\;\circ }$C) and overestimates it for small organisms at a low temperature ($10^{\;\circ }$C). However, the mismatch is not large given the scatter in the data.

**Figure 5:**
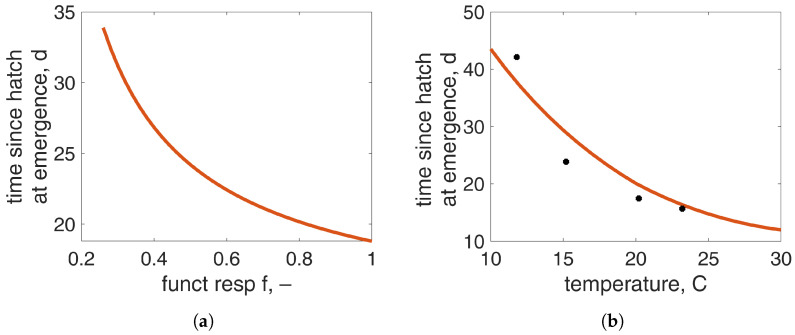
Time since hatch at emergence as function of (**a**) scaled functional response (at 20 °C) and (**b**) temperature (with f=1). The dots represent data from [39], which are not used in the parameter estimation.

Figure 5 illustrates the model’s capability to predict the time at emergence as a function of food availability and temperature. With the assumption of the second phase in the last larval instar, during which the reserves are fueled to overcome the organism’s demands during the subsequent non-feeding stages, the larval duration increases, almost doubling the time under conditions of food scarcity, which agrees with experimental evidence. Regarding the impact of temperature on time at emergence, the model within the range of temperatures of $10^{\;\circ }$C to $30^{\;\circ }$C predicts an increase in time from 12.7 days to 42.8 days, predictions that align with the data from [39]. Note that these data are only used for model validation and they have not been used in the estimation procedure. The model is less efficient in predicting the time at emergence below $15^{\;\circ }$C. To enhance model predictability at the lower part of the thermal range, the full five-parameter temperature correction function [53] will be needed. In this case, data at low temperatures are required to estimate the additional two parameters: the lower boundary of the tolerance range $T_{L}$ and the Arrhenius temperature for the rate of decrease at the lower boundary $T_{AL}$.

**Figure 6:**
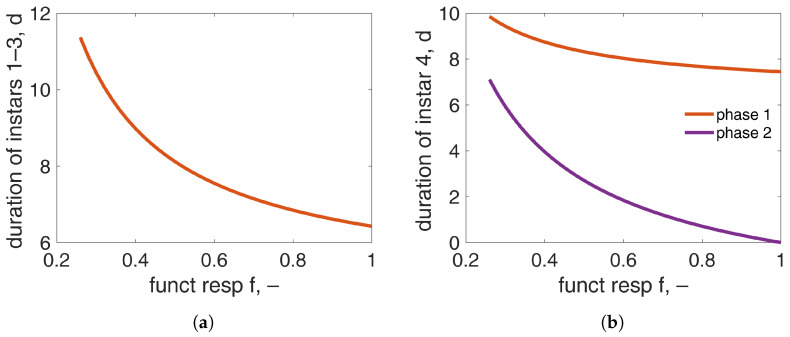
(**a**) Duration of the first three instars and (**b**) duration of Phase I (orange) and Phase II (purple) of the fourth larval stage (at 21 °C) as a function of scaled functional response.

Figure 6 demonstrates the duration of the distinct larval stages as a function of the scaled functional response. The duration of the immature stages, which include L1–L3 instars, increase by approximately five days while decreasing the scaled functional response from f=1 to the minimum feeding level that allows pupation to be reached. The duration of the L4 instar is split into Phase I and Phase II. The duration of Phase I increases by about two days between the two extreme feeding levels, while the duration of Phase II is prolonged by seven days in these simulations. In total, the duration of Phase II has a larger contribution in the overall increase in the L4 instar duration when food availability is low.

## 4. Discussion

In recent years, an increasing number of studies have used mathematical models for chironomids, particularly to assess the effects of toxicants [33,34,35,36,37,38,39]. It is therefore very essential to capture their life cycle and sensitivities to environmental factors prior to any additional stress to toxicant exposure. Although work has been conducted on modeling the life cycle of *C. riparius*, one of the most used species for water quality analysis, additional refinements are necessary to capture the observed patterns, particularly the fact that while the species reaches a similar length independent of food availability, the number of eggs differs [11]. Differences in the number of eggs in this case could only be linked to differences in energy reserves and thus to weight. In the current study, additional experiments were performed to measure both length and weight as well as reproduction data at various food levels. The weight data were very informative for both model structure and parameter estimation.

The length-at-age data from [11] and the current study indicated that the growth rate is unaffected by the different food levels during the first two–three instars. In both studies, midges were fed each day with the specified feeding level. Individuals were provided with a range of food quantities, representing a spectrum from abundant food to limited resources. In the present study, no difference in length was observed until day 5 after hatch, while in [11], the difference between experiments was around 0.6 mm on day five. Vos et al. [67] reported differences between different food types and quantities on the growth of *C. riparius* after a week. This study reported only the sizes at the end of the experiment, and thus we do not know if this pattern was repeated in these experiments. When midges were fed with TetraMin, differences were only observed at the low concentrations, while high food availabilities resulted in a saturated growth curve. Vos et al. [67] does not report how food was administered and cannot be directly comparable with that in Pery et al. [11] and the current study where food was added daily. Moreover, the food concentrations in [67] that show differences in length after 7 days are in the range 0–0.1 mg/larva/d, which are much smaller than the concentrations tested in the other studies. Numerical simulations were performed to determine if this observation was a physiological characteristic of the species or a consequence of the increased food availability compared to the organism’s needs. The simulations indicated that food uptake during the first three instars was lower than the food administered per day, even at the lowest feeding level (Figure S1 in the Supplementary Materials). Model simulations revealed that the surplus of food may be accumulated in the medium. During the fourth larval instar, food uptake increased due to the insect’s increase in size, which is when food limitation becomes apparent. If the impact of food limitation on the duration of larval instars is the aim of a study, future experiments need to take that into account. Based on these simulations, we assumed ad libitum food during the first three instars only for the experiments conducted in the present study, since this assumption was also supported by the weight data and were not available for the other studies.

While the existing hax models for *C. riparius* [39,52] and other holometabolous species (e.g., [41,68], see also [44]) assume that puberty occurs during the larval stage, no data were previously used to estimate the time at which the organism starts to allocate energy for reproduction. This resulted in an underprediction of the time at puberty. This is very essential particularly for species that do not feed in the imago stage, since all the necessary energy for reproduction is saved from the earlier stages. Based on experimental evidence observed during the fourth larval instar of *C. riparius*, differences were reported in growth between males and females [11,57] and in energy reserves [58], and the ecdysone-responsive genes were increased, indicating the induction of vitellogenin [56]. These differences between males and females indicate that the change from immature to mature larva occurs during the L4 stage. This information was used to specify the event of puberty in the last molt from L3 to L4. Many studies report differences in the fourth larval instar of most holometabolous insects, including differences in body size between males and females [69,70,71], time at emergence [72,73], number of instars [69,74,75], oxygen consumption [76], lipid content [77], and feeding behavior [78]. These observations seem to be consistent among holometabolous insects and could potentially be used in future models. Specifying the event of puberty results in achieving improved parameter identification.

**Figure 7:**
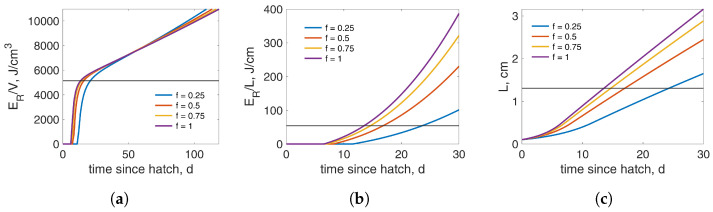
Behavior of three triggers (**a**) $E_{R}/V$, (**b**) $E_{R}/L$, and (**c**) *L* for different functional responses (f=0.25 (blue), f=0.5 (orange), f=0.75 (yellow), and f=1 (purple)). Horizontal lines denote the expected value for each trigger at pupation based on the current parameter set. If one of these triggers is used, the parameters should be re-estimated and changes are expected; however, the general behavior should remain the same. The difference on the *x*-axis of these plots was intended to show the switch in (**a**) while also showing the horizontal lines in (**b**,**c**). These plots do not incorporate Phase II of the L4 instar as it was modified in the current model.

Existing h-typified DEB models predicted a shorter duration for the larva (or nymph for hemimetabolous insects) under food limitation [53]. However, this has not been observed in many insect species (see for example [79,80,81]). Data for *C. riparius*, in particular from the current study and from [12], indicate that the duration of the fourth larval instar increases significantly with less food. This property of the h-typified DEB models was due to the assumption that the trigger for pupation (or emergence) was when the reproduction reserve density ($\frac{E_{R}}{V}$) attains a specific value ($\left[E_{R}^{j}\right]$). However, less food results in reduced growth and the threshold is reached faster. Previous attempts to address this issue have explored various triggers for pupation. Specifically, Gergs et al. [38] assumed that pupation occurs when a specific length is achieved. This does ensure that less food will result in a longer larval duration; however, the fecundity will be similar for all diets, a result which does not align with data ([11] and the current study). A more recent work by Koch et al. [39] suggested that $\frac{E_{R}}{L}$ instead of $\frac{E_{R}}{V}$ should be used as a trigger. Although this trigger results in a longer larval duration for decreasing food levels, the differences in the number of eggs for different functional responses are small. It should be noted that both studies [38,39] performed the parameter estimation with the standard $\frac{E_{R}}{V}$ trigger and, subsequently, used the resulting model to explore various triggers. This approach, however, involves inconsistencies, potentially resulting in inaccurate predictions such as the number of offspring and the effect of reduced feeding, as already discussed in [38]. It is also worth pointing out that in both versions, puberty occurred shortly after birth, which contradicts with the literature, as explained above [11,56,57,58]. Using $\frac{E_{R}}{V}=\left[E_{R}^{j}\right]$ as the trigger for pupation in combination with short immature larval duration could be the reason for underpredicting pupation at low feeding levels. The behavior of the reproduction reserve density, $\frac{E_{R}}{V}$, for various functional responses shows a characteristic change over time; a switch in the curves occurs, where they overtake each other at a specific time point, as shown in Figure 7a. This indicates that small values of $\left[E_{R}^{j}\right]$ will result in a longer duration of L4 for low feeding levels. In our proposed model with biphasic growth during the L4 instar, the switch occurs much later, and $\left[E_{R}^{j}\right]$ is attained prior to the switch. This result is probably due to the assumption that puberty occurs at the end of instar L3, which in turn ensures a smaller value of $\left[E_{R}^{j}\right]$. Therefore, by specifying puberty, the duration of the larval stage increases for lower food availability. Although the aforementioned trigger combined with a longer puberty period will result in the desired properties, this trigger alone was not sufficient to capture the prolonged duration of the L4 stage, as indicated by the data. Gergs and Baden [68], who used a hex DEB model to describe the life cycle of *Spodoptera frugiperda*, showed that the duration of the fifth larval instar increases with reduced food quality or quantity. In their study, however, the development time of each instar is defined based on Dyar’s law, which is not directly linked to the triggers for the DEB events of puberty and pupation but rather to length thresholds. Dyar’s law is an add-on to DEB models for insects and is used to indicate the beginning of each instar. Length thresholds, as discussed above, result in prolonging the duration of a stage under lower food availability, with a concurrent increase in the number of eggs. Moreover, the total duration of all instars may not always match the duration of the larval stage (from hatch to pupation) when calculated based on maturity levels and/or the reproduction reserve density triggers. This is an important concern that needs to be taken into account when both Dyar’s law and DEB-related triggers are used to calculate development times.

While the initial thought was to capture the increased duration of the larval stage by specifying the event of puberty, data from [12] and the current study show that only the duration of the last larval instar is affected by the diet, which occurs when the organism is mature. Such an increase could not be captured only by Phase I of the last larval instar (Figure 6a). Several triggers were used to resolve this issue. Figure 7b,c shows some of them. However, none were able to achieve the prolonged duration without either increasing it or resulting in small differences in the number of eggs at low food levels, as also discussed in [38,39]. Following many trials, the desired results were achieved only after incorporating an additional trigger. Specifically, in addition to the reproduction reserve density reaching a specified value, the reserve density should also assume a critical value. This is because the organism allocates energy to growth and reproduction simultaneously. Consequently, any trigger that increases the stage duration will also increase fecundity. Achieving a “critical” weight prior to pupation is common practice in many holometabolous species [82,83,84,85].

Studies in Chironomidae have shown that females need this excess energy to extend their life span while males expand their duration of flight, both increasing the chances of successful reproduction [86,87]. As pointed out in [41], requiring the reproduction reserve density $\frac{E_{R}}{V}$ to achieve a specified value helps to ensure a critical mass of the organism at pupation. However, this trigger does not take into account the energy in the general reserve. Incorporating the two triggers ensures sufficient energy for successful pupation, metamorphosis, survival, and reproduction. Strandberg et al. [88] observed that the proportion of n–6 polyunsaturated fatty acids (PUFAs), which are linked with the regulation of metabolism and the reproductive system [89], were almost independent of the diet. This observed pattern resembles the assumption of the biphasic L4 instar growth, where both the reserve and reproduction reserve density are the same at pupation under all feeding levels. However, the growth is still affected, as shown in Figure 2, Figure 3 and Figure 7a.

Servia et al. [58] also showed that the last instar can be divided into two phases for both sexes. In the first phase, the trehalose levels are almost constant, while in the latter, the levels increase rapidly. Trehalose is an instant energy source used by flight muscles and for chitin synthesis, which has been suggested to play an important part in insects’ metamorphosis [90,91]. This agrees with our modification of the DEB model, where the organism saves energy for essential processes during the subsequent non-feeding stages. Refs. [11,31] presented growth and reproduction data for different concentrations of TetraMin. In [11], only the growth was modeled, and in [31], an equation to capture the reproduction data was presented. In their studies, it was assumed that growth continued until a maximum length was reached, after which growth ceases. To capture the prolonged duration of the fourth larval instar, a delay between the time that the maximum length is reached and the time of emergence was assumed. This was modeled by using another parameter, different for each feeding level. Such an approach does not have a mechanistic basis and increases the number of parameters for each feeding level. Moreover, reproduction is related directly to food availability, and it is not linked to size and duration of feeding [31]. By not linking feeding, growth, and reproduction, the extended larval duration does not affect the reproduction, which is the reason they managed to capture both endpoints well. Moreover, they assume that since the maximum length is the same, the weight at the time that the maximum length is reached should also be the same between the experiments, and that the differences in reproduction are due to differences in the period following that. This is in contradiction with the data from the current study, where between the experiments the length is similar for an extended period, while differences in weight are noticed earlier. Therefore, it was essential to analyze weight data in addition to length data to make the most accurate assumptions about the model.

An assumption in DEB theory that is unclear whether it is true or not for insects is that the maternal diet affects the life of the offspring. Mothers raised under poor conditions will produce eggs with less reserves, which will then affect the growth of embryos and larvae. This characteristic seems to be species- or order-specific in insects. For many insect species, maternal diet affects the offspring [92,93,94]. However, for many aquatic insects, this might not be true; examples include *Chironomus tentans* [95], *Chironomus tepperi* [14], *Hexagenia limbada*, and *Hexagenia rigida* [96]. In the current study for *Chironomus riparius*, we assume that there is no maternal effect, and the egg size is independent of the mother’s condition.

The proposed DEB model with the biphasic L4 instar growth revealed that lower feeding levels and lower temperatures result in a prolonged larval duration and captured the times at life history events (pupation, emergence, etc.) and egg production well. This model has the potential to be integrated with TKTD models to study the effects of toxicants on a variety of traits, such as feeding, growth, or reproduction. The proposed modifications to the hax-typified DEB model are general, allowing it to be adapted for use by Chironomidae and other holometabolous insects that share similar life stages.

## Figures and Tables

**Table 1:** Energy fluxes linked to metabolic processes, state variables, and dynamics of the modified hax DEB model. Model parameters are described in Table 2.

**Metabolic Process**	**Energy Flux**
Assimilation	${\dot{p}}_{A}=\left\{{\dot{p}}_{Am}\right\}s_{M}fV^{2/3}$ ^(1)^
Mobilization	${\dot{p}}_{C}=E\frac{\left[E_{G}\right]\dot{v}s_{M}V^{2/3}+\left[{\dot{p}}_{M}\right]V}{\kappa E+[E_{G}]V}$ ^(2)^
Somatic maintenance	${\dot{p}}_{S}=\left[{\dot{p}}_{M}\right]V$
Growth	${\dot{p}}_{G}=\kappa {\dot{p}}_{C}-{\dot{p}}_{S}$ ^(3)^
Maturity maintenance	${\dot{p}}_{J}={\dot{k}}_{J}E_{H}$
Maturation/reproduction	${\dot{p}}_{R}=\left(1-\kappa \right){\dot{p}}_{C}-{\dot{p}}_{J}$ ^(4)^
**State Variables**	**Dynamics**
Energy in reserve, *E*	$\frac{dE}{dt}={\dot{p}}_{A}-{\dot{p}}_{C}$
Structural body volume, *V*	$\frac{dV}{dt}=\frac{{\dot{p}}_{G}}{\left[E_{G}\right]}$
Energy invested into maturation, $E_{H}$	$\frac{dE_{H}}{dt}={\dot{p}}_{R}$ ^(5)^
Energy invested into reproduction, $E_{R}$	$\frac{dE_{R}}{dt}={\dot{p}}_{R}$ ^(6)^

^(1)^ ${\dot{p}}_{A}=0$ during the egg, pupa, and imago stage. ^(2)^ ${\dot{p}}_{C}={\dot{p}}_{S}+{\dot{p}}_{J}$ during the second phase of the L4 and $E\dot{v}s_{M}V^{-1/3}$ during the imago stage. ^(3)^ ${\dot{p}}_{G}=0$ during the second phase of the L4 and imago stage. ^(4)^ ${\dot{p}}_{R}={\dot{p}}_{C}-{\dot{p}}_{S}-{\dot{p}}_{J}$ during the imago stage. ^(5)^ Investment in maturity stops at puberty ($E_{H}=E_{H}^{p}$) and begins from zero at pupation ($E_{H}=0$) until emergence ($E_{H}=E_{H}^{e}$). ^(6)^ Investment in reproduction starts at puberty ($E_{H}=E_{H}^{p}$) and stops during the L4 instar when $E_{R}/V=\left[E_{R}^{j}\right]$. Note: The acceleration factor $s_{M}$ increases with the structural length $L\equiv V^{1/3}$. Specifically, $s_{M}=\max\left(1,\min\left(L,L_{p}\right)/L_{b}\right)$, where $L_{b}$ and $L_{p}$ are the structural lengths at birth and puberty, respectively.

**Table 2:** DEB parameters of the *Chironomus riparius* model at $T_{ref}$ = 20 °C.

Symbol	Value	Units	Description
${\left\{{\dot{p}}_{Am}\right\}}^{\left(*\right)}$	203.0	${\mathrm{J/d\cdot cm}}^{2}$	Surface-area-specific maximum assimilation rate
${\dot{v}}^{\left(*\right)}$	0.007	cm/d	Energy conductance
κ	0.44	-	Allocation fraction to soma
$\left[{\dot{p}}_{M}\right]$	145.1	${\mathrm{J/d\cdot cm}}^{3}$	Volume-specific somatic maintenance
${\dot{k}}_{J}$	0.036	1/d	Maturity maintenance rate coefficient
$\left[E_{G}\right]$	4018	${\mathrm{J/cm}}^{3}$	Specific cost for structure
$E_{H}^{b}$	0.003	J	Maturity at birth
$E_{H}^{p}$	0.277	J	Maturity at puberty
$E_{H}^{e}$	1.072	J	Maturity at emergence
$\left[E_{R}^{j}\right]$	5323	${\mathrm{J/cm}}^{3}$	Reproduction buffer at pupation
$\kappa_{V}$	1.1 × $10^{-6}$	-	Conversion efficiency E→V→E
$T_{A}$	6420	K	Arrhenius temperature
$T_{AH}$	18,250	K	Arrhenius temperature for upper boundary
$T_{H}$	309.9	K	Upper boundary of tolerance range
$\delta_{M_{1}}$	0.083	-	Shape coefficient for alive individuals
$\delta_{M_{2}}$	0.067	-	Shape coefficient for dead individuals
$s_{1}$	2.48	-	Constant for 1st molt
$s_{2}$	2.48	-	Constant for 2nd molt
${\dot{h}}_{b}$	0.02	1/d	Background hazard rate coefficient
${\dot{k}}_{starv}$	3.09	1/d	Killing rate coefficient
${\left\{{\dot{p}}_{Am_{m}}\right\}}^{\left(*\right)}$	172.3	${\mathrm{J/d\cdot cm}}^{2}$	Maximum specific assimilation rate for males

^(*)^ These parameters are multiplied with the acceleration factor $s_{M}$, which ranges from 1 (until birth) and increases linearly with structural length until puberty to 4.72 at abundant food, after which it remains constant.

**Table 3:** Zero-variate data and model predictions for *Chironomus riparius*.

Data	Value	Units	Description
2.0	3.3	d	Age at birth at ${\mathrm{21}}^{\;\circ }$C ^(1)^
7.0	6.4	d	Time since birth at puberty at ${\mathrm{21}}^{\;\circ }$C ^(2)^
15.0	13.8	d	Time since birth at pupation at ${\mathrm{21}}^{\;\circ }$C ^(3)^
25.0	22.0	d	Time since birth at pupation at ${\mathrm{15}}^{\;\circ }$C ^(3)^
19.3	20.0	d	Time at emergence at ${\mathrm{20}}^{\;\circ }$C ^(4)^
0.09	0.10	cm	Length at birth ^(5)^
1.38	1.57	cm	Length at pupation ^(1)^
${\mathrm{0.99}\cdot \mathrm{10}}^{-3}$	${\mathrm{0.83}\cdot \mathrm{10}}^{-3}$	mg	Initial egg ash free dry weight ^(6)^
10	9.7	mg	Wet weight at pupation ^(7)^
1.1	1.6	mg	Dry weight of imago ^(8)^
2.0	1.9	d	Duration of instar 1 at ${\mathrm{21}}^{\;\circ }$C ^(2)^
2.0	1.9	d	Duration of instar 2 at ${\mathrm{21}}^{\;\circ }$C ^(2)^
3.0	2.6	d	Duration of instar 3 at ${\mathrm{21}}^{\;\circ }$C ^(2)^

Data sources: ^(1)^ [11], ^(2)^ [12], ^(3)^ [30], ^(4)^ [39], ^(5)^ current study, ^(6)^ [63], ^(7)^ [64], and ^(8)^ [27].

## Data Availability

The latest version of the four codes used for the DEB models are available in the Add-my-Pet library at https://www.bio.vu.nl/thb/deb/deblab/add_my_pet/entries_web/Chironomus_riparius/Chironomus_riparius_res.html (accessed on 21 September 2024).

## Supplementary Materials

The following supporting information can be downloaded at https://www.mdpi.com/article/10.3390/insects15110848/s1, Supplementary Materials associated with this article can be found in the online version and include (1) Experimental Data, (2) The DEB model, (3) Food availability, and (4) Additional results.
