# Supplementary material for: Modeling the Bioenergetics and Life History Traits of Chironomus riparius–Consequences of Food Limitation
Source: Insects. 2024 Oct 30;15(11):848. doi: 10.3390/insects15110848 (PMC11594967; doi:10.3390/insects15110848)
Supplement: Supplementary file 1 [file insects-15-00848-s001.zip › insects-3248782-supplementary.pdf]

Supplementary Material

Modeling the bioenergetics and life history traits of *Chironomus riparius* – consequences of food limitation

E. Klagkou[1], A. Gergs[2], C. U. Baden[2], K. Lika[1,*]

[1] Department of Biology, University of Crete, Heraklion 70013, Greece

[2] Bayer AG, Crop Science Division, Alfred-Nobel Straße 50, 40789 Monheim, Germany

*Corresponding author: lika@uoc.gr

1 Experimental Data

Experiments were performed, to test the influence of food availability on the growth and reproduction of *Chironomus riparius* in a static laboratory test system. Four different concentration of TetraMin® commercial fish food flakes were used in this study: 0.1, 0.15, 0.3 and 0.6 mg/larva/d. Newly hatched larvae were collected and randomly inserted into glass beakers, 20 animals in each one. The larvae were fed with the appropriate amount of TetraMin suspension every working day. Every 3-4 days, two beakers of every test concentration were rinsed out and the larvae were collected, measured and weighed. Number and gender of the emerged animals was determinated and noted. All emerged female midges per day and feeding concentration were caught with a suction aid and put together in flight cages. For every female midge, 3 male midges from the breeding culture were added for mating. The vessel filled with water inside of the flight cages serves for the egg clutches. The deposited egg clutches were removed and counted daily. When the single eggs were clearly visible, the egg clutches were laid out on a non-strongly absorbent. After 3-5 days kept in the fridge, the eggs were scattered and countable under a binocular. The counted eggs were added up for each egg clutch and recorded. Tables S1 and S2 give the body length (mm) and wet weight (mg) and Table S3 gives the time and weight at pupation and number of eggs per female at each concentration.

Table S1. Nymphal body length (mm) (mean±SD, number of individuals) under different food availability.

Time since hatch (d)	0.6 mg/larva/d	0.3 mg/larva/d	0.15 mg/larva/d	0.1 mg/larva/d
1	-	0.92 ± 0.06, n=20	0.92 ± 0.06, n=20	0.92 ± 0.06, n=20
4	-	2.48 ± 0.26, n=17	2.30 ± 0.38, n=20	2.35 ± 0.13, n=10
5	3.51 ± 0.24, n=40	-	-	-
8	6.89 ± 0.64, n=28	8.37 ± 1.05, n=36	7.33 ± 0.90, n=35	8.19 ± 0.88, n=23
10	10.87 ± 1.07, n=24	11.24 ± 1.50, n=29	9.91 ± 1.11, n=39	9.13 ± 1.51, n=33
12	13.17 ± 1.09, n=34	12.82 ± 0.78, n=42	11.51 ± 0.81, n=41	10.36 ± 0.90, n=37
15	14.49 ± 1.18, n=16	14.38 ± 1.66, n=14	14.19 ± 0.60, n=7	10.54 ± 0.79, n=23
16	-	13.54 ± 1.23, n=16	11.83 ± 0.94, n=23	11.23 ± 0.97, n=18
17	-	14.69 ± 1.32, n=9	12.58 ± 0.89, n=18	11.43 ± 0.73, n=19
18	-	-	11.74 ± 0.89, n=22	13.38 ± 1.07, n=2
19	-	-	14.22 ± 1.39, n=10	12.05 ± 0.98, n=16
22	-	-	-	12.36 ± 1.31, n=3
23	-	-	-	11.50 ± 0.78, n=18
25	-	-	-	12.55 ± 0.95, n=17
26	-	-	-	12.81 ± 0.92, n=7
29	-	-	-	13.54 ± 1.47, n=8

Table S2. Nymphal wet weight (mg) (mean±SD, number of individuals) under different food availability.

Time since hatch (d)	0.6 mg/larva/d	0.3 mg/larva/d	0.15 mg/larva/d	0.1 mg/larva/d
1	-	-	-	-
4	-	-	-	-
5	0.020 ± 0.03, n=40	-	-	-
8	1.40 ± 0.41, n=28	2.24 ± 0.71, n=35	1.54 ± 0.35, n=35	1.93 ± 0.60, n=23
10	5.19 ± 0.85, n=24	3.61 ± 1.55, n=29	2.99 ± 0.80, n=39	2.48 ± 1.08, n=33
12	7.11 ± 2.97, n=34	7.04 ± 1.33, n=40	4.75 ± 0.94, n=40	3.58 ± 0.77, n=35
15	9.47 ± 1.76, n=16	9.73 ± 2.43, n=14	7.95 ± 1.72, n=7	3.93 ± 0.74, n=23
16	-	8.13 ± 1.54, n=16	5.01 ± 1.08, n=24	4.50 ± 0.93, n=18
17	-	9.52 ± 1.75, n=9	5.60 ± 1.20, n=18	4.44 ± 0.88, n=19
18	-	-	4.99 ± 1.03, n=22	7.52 ± 1.69, n=2
19	-	-	8.59 ± 1.77, n=10	5.27 ± 1.08, n=16
22	-	-	-	4.43 ± 1.78, n=3
23	-	-	-	4.40 ± 1.17, n=18
25	-	-	-	6.08 ± 1.20, n=17
26	-	-	-	6.68 ± 1.13, n=7
29	-	-	-	6.67 ± 2.40, n=8

Table S3. Time (d), wet weight (mg) at pupation and cumulative number of eggs (mean±SD) under different food availability.

	0.6 mg/larva/d	0.3 mg/larva/d	0.15 mg/larva/d	0.1 mg/larva/d
Time at pupation (d)	15.28	17	21.15	31.4
Number of eggs/female (measured as sum of eggs/sum of clutches)	420	325	275	227

2 The DEB model

The hax typified model assumes five life stages: embryo, juvenile, adult, pupa, and imago. Table S4 presents the energy fluxes for each stage and Table S5 presents the dynamics of the four state variables (reserve E, structure volume V, energy invested into maturation E_H, and energy invested into reproduction E_R). Table S4 presents the energy fluxes for the embryo (egg), the juvenile (L1-L3 instars), the adult (L4 instar, Phase 1 and Phase 2), the pupa and imago stages. It is assumed that the immature larva (L1-L3) grows as V1-morph, while all other stages grow isomorphically. The surface-area-specific assimilation rate $\{\dot{p}_{Am}\}$ and the energy conductance $\dot{v}$ influences by this change and change values during ontogeny. Specifically, these parameters are multiplied by the acceleration factor $s_M = \max(1, \frac{\min(L, L_p)}{L_b})$, where L_b and $L_p$are the structural length at birth and puberty, respectively. All parameters are given in Table 2 of the main text.

Table S4. Energy fluxes for the egg, larva, pupa and imago stages.

Stage	Metabolic process	Energy flux
Egg	Assimilation	$\dot{p}_A = 0$
	Mobilization	$\dot{p}_C = E\dfrac{[E_G]\dot{v}V^{\frac{2}{3}} + [\dot{p}_M]V}{\kappa E + [E_G]V}$
	Somatic maintenance	$\dot{p}_S = [\dot{p}_M]V$
	Growth	$\dot{p}_G = \kappa\dot{p}_C - \dot{p}_S$
	Maturity maintenance	$\dot{p}_J = \dot{k}_J E_H$
	Maturation	$\dot{p}_R = (1-\kappa)\dot{p}_C - \dot{p}_J$
L1-L3 instars	Assimilation	$\dot{p}_A = \{\dot{p}_{Am}\}s_M V^{2/3}$
	Mobilization	$\dot{p}_C = E\dfrac{[E_G]\dot{v}s_M V^{\frac{2}{3}} + [\dot{p}_M]V}{\kappa E + [E_G]V}$
	Somatic maintenance	$\dot{p}_S = [\dot{p}_M]V$
	Growth	$\dot{p}_G = \kappa\dot{p}_C - \dot{p}_S$
	Maturity maintenance	$\dot{p}_J = \dot{k}_J E_H$
	Maturation	$\dot{p}_R = (1-\kappa)\dot{p}_C - \dot{p}_J$
L4 instar Phase I	Assimilation	$\dot{p}_A = \{\dot{p}_{Am}\}s_M V^{2/3}$
	Mobilization	$\dot{p}_C = E\dfrac{[E_G]\dot{v}s_M V^{\frac{2}{3}} + [\dot{p}_M]V}{\kappa E + [E_G]V}$
	Somatic maintenance	$\dot{p}_S = [\dot{p}_M]V$
	Growth	$\dot{p}_G = \kappa\dot{p}_C - \dot{p}_S$
	Maturity maintenance	$\dot{p}_J = \dot{k}_J E_H$
	Reproduction	$\dot{p}_R = (1-\kappa)\dot{p}_C - \dot{p}_J$
L4 instar Phase II	Assimilation	$\dot{p}_A = \{\dot{p}_{Am}\}s_M V^{2/3}$
	Mobilization	$\dot{p}_C = \dot{p}_S + \dot{p}_J$
	Somatic maintenance	$\dot{p}_S = [\dot{p}_M]V$
	Growth	$\dot{p}_G = 0$
	Maturity maintenance	$\dot{p}_J = \dot{k}_J E_H$
	Reproduction	$\dot{p}_R = 0$
Pupa	Assimilation	$\dot{p}_A = 0$
	Mobilization	$\dot{p}_C = E\dfrac{[E_G]\dot{v}s_M V^{\frac{2}{3}} + [\dot{p}_M]V}{\kappa E + [E_G]V}$
	Somatic maintenance	$\dot{p}_S = [\dot{p}_M]V$
	Growth	$\dot{p}_G = \kappa\dot{p}_C - \dot{p}_S$
	Maturity maintenance	$\dot{p}_J = \dot{k}_J E_H$
	Maturation	$\dot{p}_R = (1-\kappa)\dot{p}_C - \dot{p}_J$
Imago*	Assimilation	$\dot{p}_A = 0$
	Mobilization	$\dot{p}_C = E\dfrac{\dot{v}s_M}{V^{1/3}}$
	Somatic maintenance	$\dot{p}_S = [\dot{p}_M]V$
	Growth	$\dot{p}_G = 0$
	Maturity maintenance	$\dot{p}_J = \dot{k}_J E_H$
	Reproduction	$\dot{p}_R = \dot{p}_C - \dot{p}_S - \dot{p}_J$

* The imago dynamics are taken from [41]. In the present study this stage is not modeled as its duration lasts about one day. No additional energy is accumulated for reproduction; it uses the energy stored in the reproduction buffer to lay the eggs; so $\dot{p}_C = \dot{p}_S + \dot{p}_J$ and $\dot{p}_R = 0$.

Table S5. Dynamics of the state variable for all stages. The organism allocates energy for maturation in the egg, L1-L3 and pupa stage. In the rest of the stages, the organism is assumed mature and invests energy for reproduction.

State variables	Dynamics
Energy in reserve, E	$\frac{dE}{dt} = \dot{p}_A - \dot{p}_C$
Structural body volume, V	$\frac{dV}{dt} = \frac{\dot{p}_G}{[E_G]}$
Energy invested into maturation, E_H	$\frac{dE_H}{dt} = \begin{cases} \dot{p}_R, & E_H < E_H^p \text{ (egg, L1 − L3) or } E_H < E_H^e \text{ (pupa)} \\ 0, & \text{otherwise} \end{cases}$
Energy invested into reproduction, E_R	$\frac{dE_R}{dt} = \begin{cases} 0, & \text{otherwise} \\ \dot{p}_R, & E_H \geq E_H^p \text{ and } \frac{E_R}{V} < [E_R^j](L4\ phase\ I) \end{cases}$

To account for temperature effects, all rate parameters in the model ($\{\dot{p}_{Am}\}$, $\dot{v}$, $[\dot{p}_M], \dot{k}_J, \dot{h}_b$) are multiplied by the temperature correction factor c_T, including the Weibull aging acceleration, $\ddot{h}_a$, which is multiplied by c_T^2. The 3-parameter temperature correction factor is given by

$$c_T = \begin{cases} exp\left(\frac{T_A}{T_{ref}} - \frac{T_A}{T}\right), & T < T_{ref} \\ exp\left(\frac{T_A}{T_{ref}} - \frac{T_A}{T}\right)\frac{1 + exp\left(\frac{T_{AH}}{T_H} - \frac{T_{AH}}{T_{ref}}\right)}{1 + exp\left(\frac{T_{AH}}{T_H} - \frac{T_{AH}}{T}\right)}, & T > T_{ref} \end{cases}$$

where T_{ref} is the reference temperature (293.15 K or 20°C by convention), T_A the Arrhenius temperature, T_H the upper boundary of the optimal thermal range, and T_{AH} the Arrhenius temperature for the rate of decrease at the upper boundary. We used the upper boundary 3-parameter correction factor, because we could estimate the upper limits with the available temperature dependent data. The full 5-parameter correction factor could not be determined by the available data. The temperature correction is coded by the `tempcorr.m` DEBtool function.

3 Food availability

3.1 *Functional response for the data of this study*

The growth rate of the first three instars seemed to be unaffected by the food availability, as shown the length-at-age data (Table S1). This could either be because the food was abundant at all food levels or it is an inherent physiological trait of the species. To elucidate the cause, we tested the following types of functional response and computed the food availability during the experiment (input-output):

A) A constant f for each experiment (estimated value)
B) Constant $f = 1$ until puberty for all experiments, linearly decreasing till an estimated value (f_{min}) at a specific time much earlier than pupation, then remaining constant at f_{min}

In Figure S1, 1st row, the excess food is the cumulative food added in the experiment minus the food eaten by the organism. Positive values of excess food indicate that there is enough food, resulting in no growth limitation. In this theoretical experiment the dynamics of food was not modeled, since that would require data on food consumed by the organism to estimate the half saturation constant. Losses due to water renewals or food degradation were not considered. In both scenarios during the first few days, the food was abundant even in the lower food concentration (Figure S1, 1st row). Specifically, in scenario B we observed stress at the lowest food concentration on day 8, which is approximately the duration of the first three instars. Weight-at-age and length-at-age curves (Figure S1, 2nd and 3rd rows) show clearly that in scenario A the model under-predicts initially both the weight and the length, particular in the lower food levels. On the other hand, a time-variable functional response (scenario B) captures well the growth throughout the entire larval stage. At the high food levels both scenarios work well. These simulations indicate that the organism has more available food than provided the first few days after hatch, following with increased energy requirements the first days after puberty for growth, maintenance and saving energy for reproduction. While after that, its energy requirements decrease, and the organism can survive on less food. While this type of functional response complicated the modelling and estimation procedure, it reflects more accurately the reality, since the organism's needs vary depending on the life stage. The choice of puberty as the point of decrease in food availability was made mainly to avoid increasing the parameters to be estimated since in the current study that point was very close to the time of puberty.

In these numerical experiments, we based our conclusions on the prediction for the weight rather than that of the length. Weight measurements give more information throughout the larval stage while length measurements on this species only give information on the initial phase of growth since length ceases to increase and we cannot deduce anything about the food availability. On the contrary, weight measurements also show that the initial growth remains the same for all treatments, but then they increase very fast for a few days at different rates, and finally they continue to increase linearly. Moreover, larva does not easily remain straight, making it challenging to measure its length, while its weight can be measured more accurately.

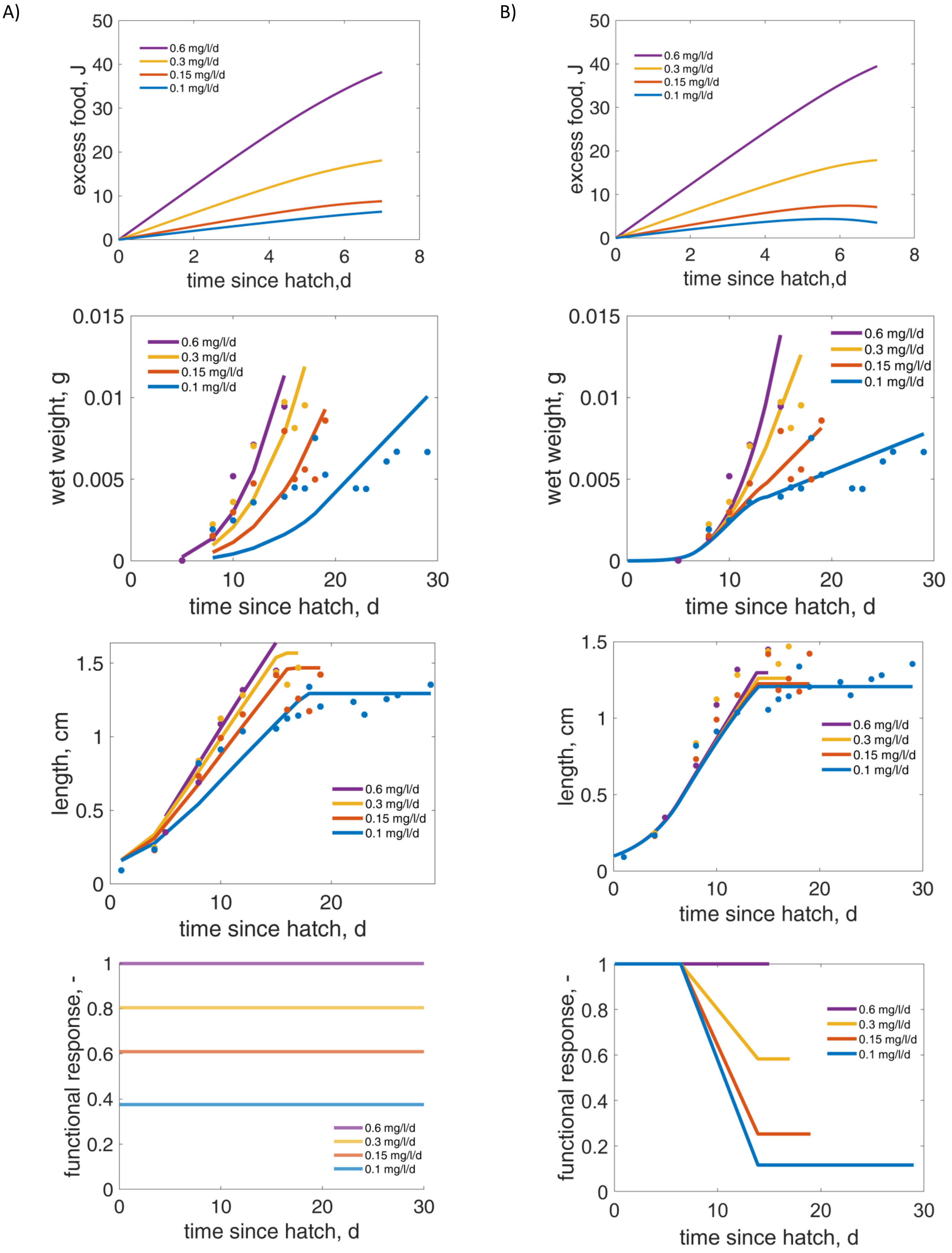

Figure S1: Excess food (first row), wet weight-at-age (second row), length-at-age (third row), and functional response (fourth row) at different food levels. Each column presents a different functional response scenario. Food was added 5/7 days a week and Tetramin is 14.4 J/mg [7].

3.1 *Functional response for the Pery at al. (2002) data*

The experimental procedure in [11] was similar with the one in the current study. Data show that growth (in terms of length) is also similar in the first days (Figure S2). However, differences occur much earlier than those in the current study. When a similar time-variable functional response, as explained in section 3.1 (scenario B), is used, initial growth is predicted well, but the length-at-age curves are very similar at all feeding levels. This was expected since puberty in that study occurred earlier than the current study, meaning that the differences are also noticeable earlier. To keep a similar scheme for the functional response, we ran estimations assuming two additional parameters, one to denote the time the functional response starts reducing (for the current study this was at puberty) and one for time when f_{min} is reached. The time parameters are the same for all feeding levels, but the f_{min} differs between them. The best fit (Figure S2a) is obtained when the functional response starts decreasing two days after birth and reached f_{min} two days later. The final values of the functional responses for each experiment (0.4, 0.3, 0.2 and 0.1 mg/larva/d TetraMin) were estimated to be 1.22, 0.85, 0.57, and 0.36, respectively. Since the functional response reached the f_{min} in four days, a constant functional response for each feeding level would also be an option. The length predictions for a time-variant functional response (Figure S2a) and a constant one (Figure S2b) are very similar indeed. Therefore, for simplicity we have used a constant functional response for each experiment; the estimated values are 1.23, 0.95, 0.76, 0.46, respectively, for each experiment (0.4, 0.3, 0.2 and 0.1 mg/larva/d TetraMin).

These additional experimental scenarios indicate that although the experiments of the current study and that of [11] were intending to be similar, details of the experimental set up such as the test vessels may produce the observed difference.

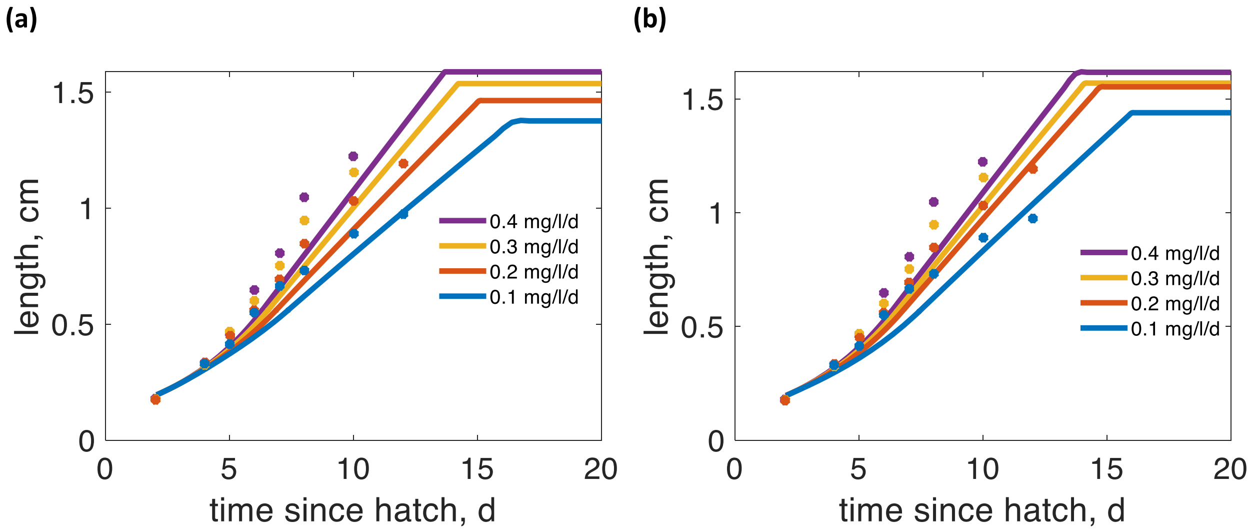

Figure S2: Length-at-age data (points) and model predictions (lines) for different concentrations of TetraMin using (a) time-variable functional response and (b) constant functional response. Data from [11].

4 Additional results

Additional data used in the parameter estimation and model predictions from the proposed model for *Chironomus riparius* are presented in Figure S3. Figure S3a presents the survival under *ad libitum* and starved feeding conditions. The probability of an individual to die, called hazard rate $\dot{h}$ is the sum of a background hazard rate $\dot{h}_b$ and a hazard rate linked with the reduction of energy reserves $\dot{h}_e$. Specifically, we assume that there is a threshold reserve energy for effects of the second hazard rate. This threshold is taken to be the maximum reserve energy at hatch: $E_{crit} = [E_m]L_b^3$. The hazard rate with energy threshold E_{crit} is defined as $\dot{h} = \dot{h}_b + \dot{k}_{starv} \max\left(0, 1 - \frac{E}{E_{crit}}\right)$, where $\dot{k}_{starv}$ is the killing rate due to starvation. Figure S3b presents the oxygen consumption as function of dry weight for two temperatures. Oxygen consumption has contribution from assimilation, growth and dissipation, the latter includes somatic and maturity maintenance and maturation. Initially the organism consumes more oxygen, which is related to its high energy needs (acceleration period). After moving to the second phase of L4 instar, the energy needs drop significantly and therefore the oxygen consumption reduces. Figure S3c presents the relation between the length and the dry weight during the larval stage. In all cases the predictions match the data very accurately.

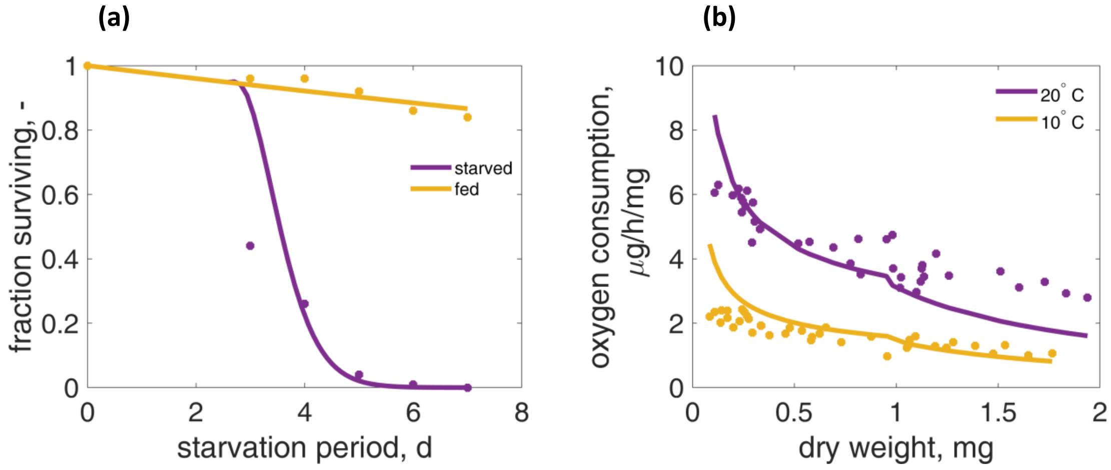

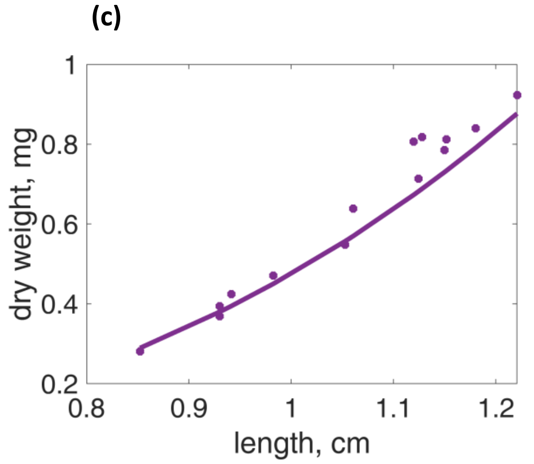

Figure S3: Data and model predictions for *C. riparius*. (a) Surviving fraction for starved and fed animals [52], (b) oxygen consumption in relation to dry weight [62] and (c) length and dry weight relationship [11].

References

[7] Habashy M. Culture of Chironomid larvae (Insecta-Diptera Chironomidae) under different feeding systems Egypt. J. Aquat. Res. **2005**, 31, 403-418.

[11] Pery, A.; Mons, R.; Flammarion, P.; Lagadic, L.; Garric, J. A modeling approach to link food availability, growth, emergence and reproduction for the midge *Chironomus riparius*. Environ. Toxicol. Chem. **2002**, 21, 2507–2513. https://doi.org/10.1002/etc.562021.

[41] Llandres, A.; Marques, G.; Maino, J.; Kooijman, S.A.L.M.; Kearney, M.; Casas, J. A dynamic energy budget for the whole life-cycle of holometabolous insects. Ecol. Monogr. **2015**, 85, 353–371. https://doi.org/10.1890/14-0976.1.

[52] Augustine, S.; Gergs, A.; AmP *Chironomus riparius* Version 2019/09/21. Available online at https://www.bio.vu.nl/thb/deb/deblab/add_my_pet/entries_web/Chironomus_riparius/Chironomus_riparius_res.html (accessed on 14 October 2024).

[62] Edwards, R.W. The relation of oxygen consumption to the body size and to temperature in the larvae of *Chironomus riparius* Meigen. J. Exp. Biol. **1956**, 35, 383-395
